# Monobac System–A Single Baculovirus for the Production of rAAV

**DOI:** 10.3390/microorganisms9091799

**Published:** 2021-08-24

**Authors:** Lionel Galibert, Aurélien Jacob, Adrien Savy, Yohann Dickx, Delphine Bonnin, Christophe Lecomte, Lise Rivollet, Peggy Sanatine, Marjorie Boutin Fontaine, Christine Le Bec, Otto-Wilhelm Merten

**Affiliations:** 1 Genethon, 1bis rue de l’Internationale, 91002 Evry, France; jacob.aurelien@hsr.it (A.J.); adrien.savy@supbiotech.fr (A.S.); ydickx@genethon.fr (Y.D.); bonnin@genethon.fr (D.B.); lecomte@genethon.fr (C.L.); rivollet.lise@uqam.ca (L.R.); psanatine@genethon.fr (P.S.); marjorie.boutin.fontaine@gmail.com (M.B.F.); christine.lebec@sensorion-pharma.com (C.L.B.); ottom@miltenyibiotec.fr (O.-W.M.); 2 Kuopio Center for Gene and Cell Therapy, Microkatu 1, 70210 Kuopio, Finland; 3 San Raffaele Telethon Institute for Gene Therapy, Via Olgettina 58, 20132 Milan, Italy; 4 Département des Sciences Biologiques, Université du Québec à Montréal, 405 rue Sainte-Catherine Est, Montréal, QC H2L 2C4, Canada; 5 Sensorion-Pharma, 375 rue du Professeur Joseph Blayac, 34080 Montpellier, France; 6 Miltenyi Biotech Europe, 10 rue Mercoeur, 75011 Paris, France

**Keywords:** rAAV, baculovirus, gene therapy

## Abstract

Large-scale manufacturing of rAAV is a bottleneck for the development of genetic disease treatments. The baculovirus/Sf9 cell system underpins the first rAAV treatment approved by EMA and remains one of the most advanced platforms for rAAV manufacturing. Despite early successes, rAAV is still a complex biomaterial to produce. Efficient production of the recombinant viral vector requires that AAV replicase and capsid genes be co-located with the recombinant AAV genome. Here, we present the Monobac system, a singular, modified baculovirus genome that contains all of these functions. To assess the relative yields between the dual baculovirus and Monobac systems, we prepared each system with a transgene encoding γSGC and evaluated vectors’ potency in vivo. Our results show that rAAV production using the Monobac system not only yields higher titers of rAAV vector but also a lower amount of DNA contamination from baculovirus.

## 1. Introduction

Adeno-associated virus is a member of the Dependovirus genus of the subfamily Parvovirinae and requires functions from helper viruses such as adenovirus [1], herpes simplex virus [2], vaccinia virus [3], or human bocavirus [4] in order to perform its replication cycle. The wild type (wt) AAV2 encodes an ssDNA genome of 4679 bases where both negative and positive strands are equally packaged in the icosahedral capsid [5]. The genome is flanked at both ends by 145 base T-shape structures called inverted terminal repeats (ITRs). These structures are necessary for AAV genome replication, second-strand synthesis [6,7], encapsidation [8], and insertion of the wt viral genome in human chromosome 19 [9]. In the recombinant AAV vector, the ITRs are flanking the recombinant transgene cassette, which allows for its replication and packaging. The ITRs also aid in the long-term maintenance of the transgene following delivery [10]. The wt-AAV2 genome encodes four replicase proteins, three capsid proteins, the membrane-associated accessory protein (MAAP) [11] and the assembly activating protein (AAP) [12].

Gene therapy using recombinant adeno-associated virus (rAAV) vector is an effective way to treat monogenic diseases. Promising results have resulted from clinical trials providing functional copies of relevant genes to patients with leber congenital amaurosis [13,14] and Hemophilia B [15].

Although expression platforms are usually trade secrets, most rAAV production systems use a variety of co-infecting AAV helper viruses. The adenoviral system has been developed and improved in terms of safety by using only essential sets of “early” adenovirus genes (e.g., E1, E2, E4, and VA RNA I and II), expressed from plasmid, without the lytic “late” genes [15,16,17,18]. In this set-up, the E1 gene is generally supplied by a producer cell line (HEK293, PER.C6, Hela-E1). Replication-defective herpes simplex virus has been used to bootstrap recombinant AAV by replacing the HSV ICP27 gene with AAV *rep* and *cap*. In turn, this deficient HSV is generated by cells supplying the ICP27 gene in trans [16].

Baculovirus-based rAAV production systems have also been used to great effect. This system uses three baculoviruses to collocate into the Sf9 cell the AAV *rep* and *cap* genes along with the rAAV transgene [17]. A significant problem with this early work was the inability of the Sf9 cell to recognize the splicing signal used by AAV [18]. This meant that the regulation of VP1-VP2-VP3 ratios was solely achieved through a ribosome scanning mechanism, linked to the use of non-ATG initiation codons. As a result, the three baculovirus system produced viral particles with a lower level of VP1 in the capsid than in the wild type. This deficiency was corrected by optimizing the Kozak sequence [19] and the start codon of VP1. These advancements were first implemented by uniQure (Patent WO2007046703) and later in the second generation system in the group of R. Kotin [20]. The use of the ribosome leaky mechanism was also used to improve Rep protein expression while at the same time improving the genetic stability of the baculovirus encoding the *rep* gene [20].

H. Chen et al. were able to eschew the ribosome scanning mechanism entirely by replacing the rAAV splicing signal with one that is functional in insect cells. Using the native splicing, they were able to efficiently produce both the large and small Rep proteins as well as the VP1-2-3 proteins [21]. They also managed to place a second promoter within the intron allowing for the expression of a second transcript (e.g., the rep52 or the vp2-3 transcript). The non-spliced version of the “cap-in” or “rep-in” contains a stop codon in frame with either the N-terminal part of VP1 or the Rep78 protein. Stable Sf9-based cell lines encoding the *rep* and *cap* genes have also been engineered [22,23]. Upon infection with the baculovirus encoding the rAAV genome, the immediate-early genes encoded by the baculovirus allow the amplification and higher expression levels of the Sf9 cell-encoded *rep* and *cap* genes due to the presence of the baculoviral cis-acting element hr2.

In this work, we developed a single baculovirus containing all the functions required to produce rAAV, the AAV *rep* and *cap* genes along with the recombinant AAV genome. This system, termed “Monobac,” has been used for the production of rAAV8 encoding a human gamma-sarcoglycan (γSGC) transgene. Vector productivity was doubled compared to the dual baculovirus system [20], while decreasing the baculovirus DNA contamination. Furthermore, in vivo injection of rAAV8-γSGC in a γSGC mouse model resulted in efficient γSGC protein expression in striated and cardiac muscle fibers.

## 2. Materials and Methods

### 2.1. Recombination of rep2-cap8 and cap8 Expression Cassettes at Bacmid egt Locus of AcMNPV Bacmid

Chloramphenicol acetyl transferase (CAT) gene flanked by lox66/lox72 sequences [24] was PCR amplified from pcrTOPO-lox-CAT-lox plasmid [25] and cloned in pFBD-rep2-cap8 plasmid [20] at unique cutter restriction site SnaBI resulting in pFBD-rep2-cap8-lox66-CAT-lox71. This plasmid was then used to amplify by PCR rep2-cap8 and cap8 expression cassettes including lox66-CAT gene-lox72 sequences using, respectively, primer pairs EGT-lox-F/EGT-SV40-R for rep2-cap8 and EGT-lox-F/EGT-p10-R for cap8 expression cassettes (primer sequences can be found in Table 1). Insertion at *egt* locus in *Autographa californica* multiple nuclear polyhedrosis virus (AcMNPV) bacmid DNA [26] was performed according to Noad et al. [27] in an *E. coli* DH10B strain containing both AcMNPV bacmid and pKD46 plasmid [28] then assessed by PCR using primers EGT-F and EGT-R.

### 2.2. Insertion of AAV rep2-cap8 Genes and Recombinant AAV Genomes into Bacmid by Transposition at Tn7 Site

rAAV sequence encoding γSGC was previously described in Herson et al. [29] and sub-cloned in pFBDual plasmid (Invitrogen) at SnaBI-StuI sites. The size of the rAAV genome cassette is 3823 bp. pFBD-Rep2-Cap8 originating from R. Kotin’s laboratory [20] was recombined in *E. coli* DH10bac containing *Autographa californica* multiple nuclear polyhedrosis virus (AcMNPV) bacmid [26]. pFBDual encoding rAAV-γSGC genome was recombined at Tn7 site of Monobac rep2-cap8 bacmid genome in *E. coli* DH10B transformed with pMON7124 plasmid accordingly to Luckow et al. [26]. Efficient recombination into bacmid genomes was assessed by PCR using primer pairs M13pucF/R and M13pucF/genta (Table 1).

### 2.3. Insertion of GFP and YFP into Bacmid by Transposition at Tn7 Site

OpIE2-GFP and Tn7 L and R recombination sequences were synthesized by Genewiz. OpIE2-YFP with Tn7 L and R recombination was then obtained by directed mutagenesis. OpIE2-YFP recombination in the Tn7 site in the Monobac-rep2-cap8 bacmid was performed as described in the previous chapter. The OpIE2-GFP sequence was inserted by the restriction cloning method to plasmid pFBD-γSGC, which was inserted in the bacmid genome by Tn7 transposition. The resulting baculoviruses were used to infect Sf9 cells at a density of 1 × 10^6^ cells mL^−1^ using an MOI of 0.05 (PFU titers) for the baculovirus encoding GFP and 0.05 for the baculovirus encoding YFP. Cell cultures were performed at a volume of 70 mL and maintained at 27 °C in 250 mL disposable shake flasks at 160 rpm. Cell samples of 1 mL were withdrawn from the culture at each time point and analyzed by flow cytometry. The co-infection process was monitored by spectral flow cytometry. The cell samples were analyzed on a spectral analyzer SP6800 (Sony Biotechnology, Weybridge, Surrey, UK). The SP6800 was equipped with three lasers: 405 nm, 488 nm, and 633 nm with 40 mW, 40 mW, and 60 mW, respectively. This instrument was equipped with a 32-channel photomultiplier tube (PMT) for a 300 nm range (500 nm to 800 nm), and two PMTs (420–440 nm and 450–470 nm). Only the 488 nm laser was used for the experiments [30]. The reference spectra of fluorochromes were used for the unmixing calculation. A reference spectrum of cellular autofluorescence was obtained from unstained cells infected by a bac-rAAV encoding γSGC transgene. Spectral analysis can both recognize autofluorescence as a specific signal and as a defined color, to provide accurate results. Spectral deconvolution (unmixing calculation) was carried out using SP6800 software (1.6.1.7136 version). All data were exported as FCS3.0 and analyzed using Kaluza Flow Cytometry software 1.2 version (Beckman Coulter, Brea, CA, USA).

### 2.4. Cell Line, Baculovirus, and rAAV Production

Sf9 cells (Gibco) were grown in suspension culture at 27 °C in SFM900III medium (Invitrogen) in 1 L Erlenmeyer flasks (Corning #431147). Baculoviruses were generated by transfection of purified bacmid DNA using Cellfectin II transfection reagent (Invitrogen) according to the guidelines of the Bac-to-Bac protocol. Clonal selection was performed using plaque assay, and baculovirus clones were amplified first on adherent Sf9 cells cultivated in 25 cm^2^ T-Flasks and then in suspension cultures of Sf9 cells in 250 mL Erlenmeyer flasks (Corning #431144). rAAV8 productions were performed by dual infection of baculoviruses harboring the recombinant AAV genome γSGC and AAV rep2-cap8 genes cloned either at the Tn7 site of the bacmids or at the *egt* locus. Baculovirus MOI of 0.05 (PFU titer) was used for each virus for dual infections with a total MOI of 0.1. An MOI of 0.1 was used with the Monobac system. Infections were performed in 70 mL of Sf9 cell culture seeded at 1 × 10^6^ cells mL^−1^ in 250 mL Erlenmeyer flasks. At 96 h post-infection, 1 mL of the total culture was sampled for direct quantification of rAAV production prior to harvest and purification. Production of rAAV8 performed in bioreactors was carried out in Sartorius 2 L glass bioreactors. Briefly, Sf9 cells were seeded at 3 × 10^5^ cells mL^−1^. Three days later, when the cell density ranged between 1 and 2 × 10^6^ cells mL^−1^, baculovirus infection was performed as described above.

### 2.5. rAAV Purification and Characterization

Seventy mL of baculovirus-infected Sf9 cultures producing rAAV8-γSGC were treated with 0.5% Triton-X-100 and Benzonase for 2.5 h at 27 °C. Treated cell culture bulks were then filtered on a Sartobran 300 0.45–0.2 µm filter (Sartorius 5231307H5). Clarified bulk cultures were purified by immuno-affinity on 50 mL of AVB sepharose medium (GE Healthcare) according to Smith et al. [23]. Purified rAAV vectors were dialyzed against PBS (Gibco) on Millipore Ultracel 100 KDa units (UFC910024). Purified rAAV vectors used for in vivo study were dialyzed against Ringer Lactate solution.

### 2.6. Determination of rAAV Genome Titer

Viral DNA was extracted directly from bulk cultures or from purified samples using MagNA Pure DNA and a viral NA small volume kit (MagNA Pure 96, Roche, Basel, Switzerland). qPCR titrations were performed using a reference (linearized plasmid) for absolute quantification with primers and probe ITR-F/R/P (Table 1) for rAAV genome titration on a Roche LightCycler 480II. Quantification of baculovirus genome copy numbers or viral titers was performed likewise using primers and probe BAC-F/R/P-Rep52-F/R/P-Cap8-F/R/P (Table 1).

### 2.7. SDS-PAGE and Western Blot

Purified rAAV vectors were run on SDS-PAGE Bis-Tris 4–12% gels (Nu-PAGE, Invitrogen, Waltham, MA, USA) and either directly Coomassie-stained or transferred on nitrocellulose membrane (iBlot gel transfer stack nitrocellulose, Invitrogen, Waltham, MA, USA) prior to Western blot immunodetection. Anti-VP primary antibody mouse igG1 clone B1 (Progen) was used at a 1/250th dilution. The secondary antibody was goat anti-mouse dye 680 (LI-COR) used at a 1/5000th dilution. Hybridization was performed in infrared imaging system blocking buffer (LI-COR), and revelation was performed on an Odyssey system (LI-COR).

### 2.8. Analytical Ultra Centrifugation

Analytical ultracentrifugation [31] was performed in a ProteomeLab XL-1 centrifuge (Beckman) using an AN-60TI rotor (Beckman) at 20 °C. Four hundred µL of purified rAAV8-γSGC were loaded into each sample cell. A first set of runs (absorbance and interference measure) was performed at 3000 rpm in order to set the wavelength of analysis and the laser position properly. Absorbance and interference monitoring were performed at 16,000 rpm from 2 h to 2 h 30 min. Data were collected using ProteomeLab software (Beckman) and treated with Sedfit software. Runs were performed against a reference rAAV8 production produced without any transgene.

### 2.9. In Vivo Injection, Sample Collection, and Gamma Sarcoglycan Protein Detection

Male HO * HO gamma sarcoglycan Balb/c mice [32], at 4 weeks old, were anesthetized by intraperitoneal injection of 100 mg of ketamine and 10 mg of xylazine per kilogram of body weight. 5 × 10^12^ vg kg^−1^ of rAAV8-γSGC in Ringer Lactate buffer, corresponding to a volume of 500 μL for a weight of 20 g, were intravenously injected in the tail vein. Five mice per group were injected using rAAV8-γSGC produced either with dual baculovirus or Monobac systems. One mouse was injected as a control with Ringer lactate buffer alone. Four weeks post-injection, mice were sacrificed by cervical elongation. Heart, loge anterior, quadriceps, psoas, deltoid, diaphragm muscles were harvested, frozen in nitrogen-cooled isopentane, and transferred to liquid nitrogen prior to histological work. Eight μm muscle sections were cut, fixed in 0.5% glutaraldehyde, and then washed twice with PBS. Fifty μL of primary antibody NCL-g-SARC (Leica Biosystems) was applied to the muscle sections, incubated for 1 h at 25 °C, and washed 3 times in TBS buffer. Then, 50 μL of anti-rabbit secondary antibody conjugated to peroxidase was applied to muscle sections and incubated for 1 h at 25 °C, washed 3 times in TBS buffer, and followed by 3,3’-diaminobenzidine revelation and light microscopy analysis. Muscle integrity was evaluated by hematoxylin-eosin staining and light microscopy analysis. The percentage of positive gamma-sarc muscle fibre sections was then quantified. All mice were handled according to directive 2010/63/EU on the protection of animals used for scientific purposes.

## 3. Results

### 3.1. Production of rAAV Using Dual Baculovirus System Encoding Fluorescent Protein Genes

The production of rAAV8-γSGC was performed by co-infecting Sf9 cells with a baculovirus encoding the AAV *rep2* and *cap8* genes, which are controlled by the very-late polh and p10 promoters, respectively, and with a baculovirus encoding the rAAV-γSGC transgene. To monitor the kinetics of infection of those baculoviruses during the rAAV production, we cloned a YFP expression cassette in the baculovirus encoding the rep2-cap8 genes and a GFP expression cassette in the baculovirus encoding the rAAV-γSGC transgene. This setup allowed us to monitor the proportion of cells that were infected with one, both, or no baculoviruses. We also quantified the rAAV-γSGC yield and compared those results with the reference, dual-baculovirus production system.

**Figure 1:**
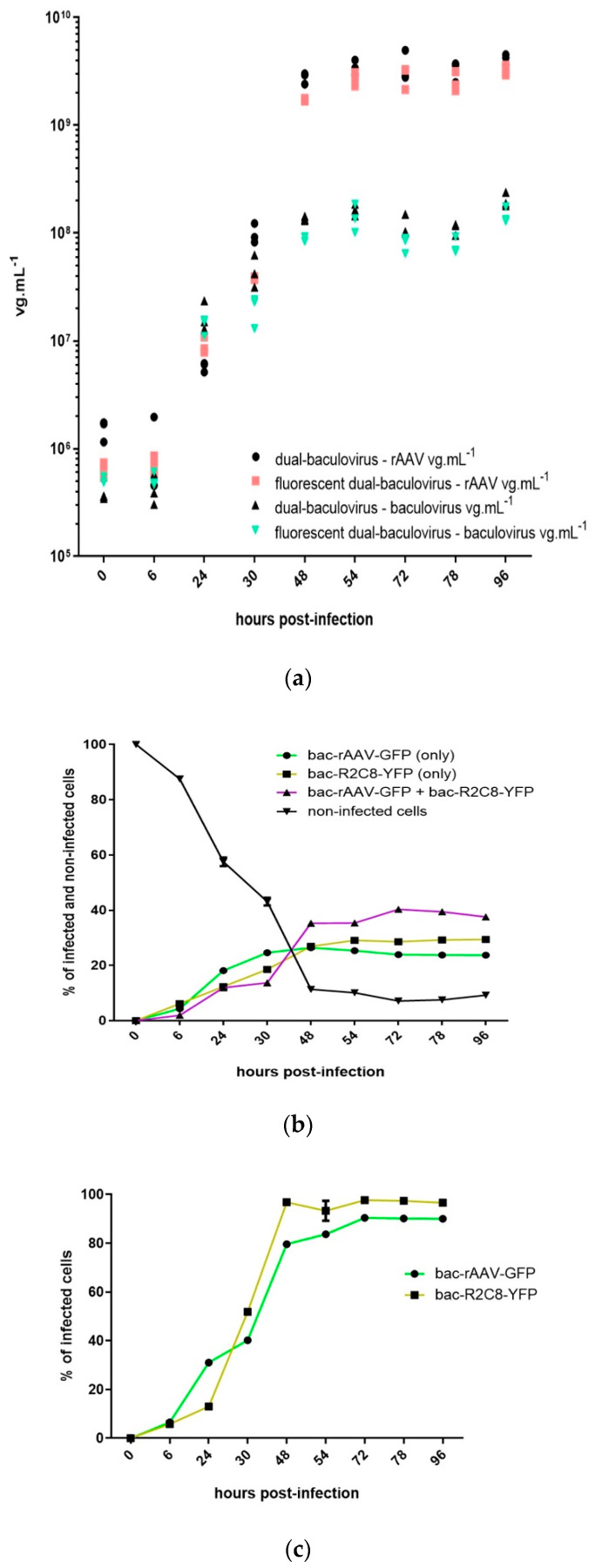
(**a**) Kinetics of production of rAAV8-γSGC and of baculovirus amplification, a comparison of dual baculovirus production system with and without reporter genes. Production of rAAV was performed by the co-infection of Sf9 cells with baculoviruses bac-rAAV-γSGC-GFP and bac-R2C8-YFP or with bac-rAAV-γSGC and bac-R2C8. Infections were performed at a MOI of 0.05 per baculovirus. rAAV titers, based on ITR amplicon qPCR, and baculovirus titers, based on DNApol amplicon qPCR, were determined at time points 0 h, 6 h, 24 h, 30 h, 48 h, 54 h, 72 h, 78 h, and 96 h post-infection. Titers of bulk samples were presented on a logarithmic scale and expressed as vg mL^−1^. “Dual-baculovirus-rAAV vg mL^−1^” represents the rAAV8- γSGC titers obtained from the co-infection performed with bac-rAAV-γSGC and bac-R2C8. “Fluorescent dual-baculovirus-rAAV vg mL^−1^” represents the rAAV titers obtained from the co-infection performed with bac-rAAV-γSGC-GFP and bBac-R2C8-YFP. “Dual-baculovirus-baculovirus vg mL^−1^” titer represents the baculovirus titers obtained from the co-infection performed with baculoviruses bac-rAAV-γSGC and bac-R2C8. “Fluorescent dual-baculovirus-baculovirus vg mL^−1^” represents the baculovirus titers obtained from the co-infection performed with baculoviruses bac-rAAV-γSGC-GFP and bac-R2C8-YFP. (**b**) Kinetics of baculovirus co-infection during rAAV production. Percentage of infection of Sf9 cells determined by FACS, during rAAV8-γSGC production, with baculovirus bac-R2C8-YFP, encoding the rep2-cap8 expression cassette and the YFP reporter gene, and baculovirus bac-rAAV-γSGC-GFP. FACS determination of the percentage of cells co-expressing GFP and YFP reporter genes, cells expressing only one of the fluorescent proteins representing single infection only with bac-rAAV-γSGC-GFP or bac-R2C8-YFP, and cells negative for both fluorescent markers. Time points were 0 h, 6 h, 24 h, 30 h, 48 h, 54 h, 72 h, 78 h, and 96 h post-infection. (**c**) Kinetics of single infection with baculovirus encoding the rAAV-γSGC and baculovirus encoding rep2-cap8 expression cassette. Percentage of infection of Sf9 cells determined by FACS during single infection performed with bac-rAAV-γSGC-GFP and bac-R2C8.

We first measured the baculovirus and rAAV titers (Figure 1a), both in the reference dual baculovirus system and one encoding the reporter genes. In general, the rAAV production increased until around 48 h post-infection before reaching a plateau. At 96 h post-infection, we measured rAAV titers of 4.13 × 10^9^ vg mL^−1^ for the reporter-less dual baculovirus production system, while we measured rAAV titers of 3.20 × 10^9^ vg mL^−1^ for the YFP + GFP dual baculovirus system. Again, baculovirus titers reached a plateau around 48 h post-infection. At 96 h post-infection, we measured baculovirus titers of 2.02 × 10^8^ vg mL^−1^ for the dual baculovirus production system without the reporter genes and 1.46 × 10^8^ vg mL^−1^ for the dual baculovirus encoding the YFP and the GFP reporter genes. These results, both in terms of rAAV titers and baculovirus titers, suggest a comparable ability to amplify the baculoviruses and produce rAAV-γSGC, in the absence or presence of the reporter genes.

We then used the YFP and GFP reporter genes encoded by the bac-R2C8 and the bac-rAAV-γSGC to monitor the infection of the Sf9 cells (Figure 1b). Forty-eight h post-infection, infection levels reach a plateau. We measured at the 96 h time point that 37.6% of the cells co-expressed both GFP and YFP, while 23.8% of cells expressed only the GFP encoded by the bac-rAAV-γSGC and 29.4% of the cells expressed only the YFP encoded by the bac-R2C8. The percentages of cells uninfected was 9.3% at the 96 h time point. Single infections led to 96.6% of the cells infected with the bac-R2C8-YFP and to 90.1% of cells infected by the bac-rAAV-γSGC-GFP at the 96 h time point (Figure 1c). These results highlight that only less than 40% of the cells were co-infected with the two baculoviruses when a total MOI of 0.1 is used, indicating that 60% of the cells will not be producing functional rAAV. Twenty-three percent of the cells infected only with the bac-R2C8 may lead to the production of capsids, potentially filled with contaminating DNA. The single infections by each baculovirus indicate that more than 90% of the cells can be infected. Thus, the development of a single baculovirus for the production of rAAV could potentially lead to higher rAAV production titers due to the higher percentage of cells infected by a single baculovirus. It would also reduce the levels of rAAV particles not filled with the rAAV transgene, as no cells would be infected only with a baculovirus encoding only the rep and cap sequences without the rAAV transgene.

### 3.2. Kinetic rAAV Genome Replication in Comparison to the Baculovirus Replication

**Figure 2:**
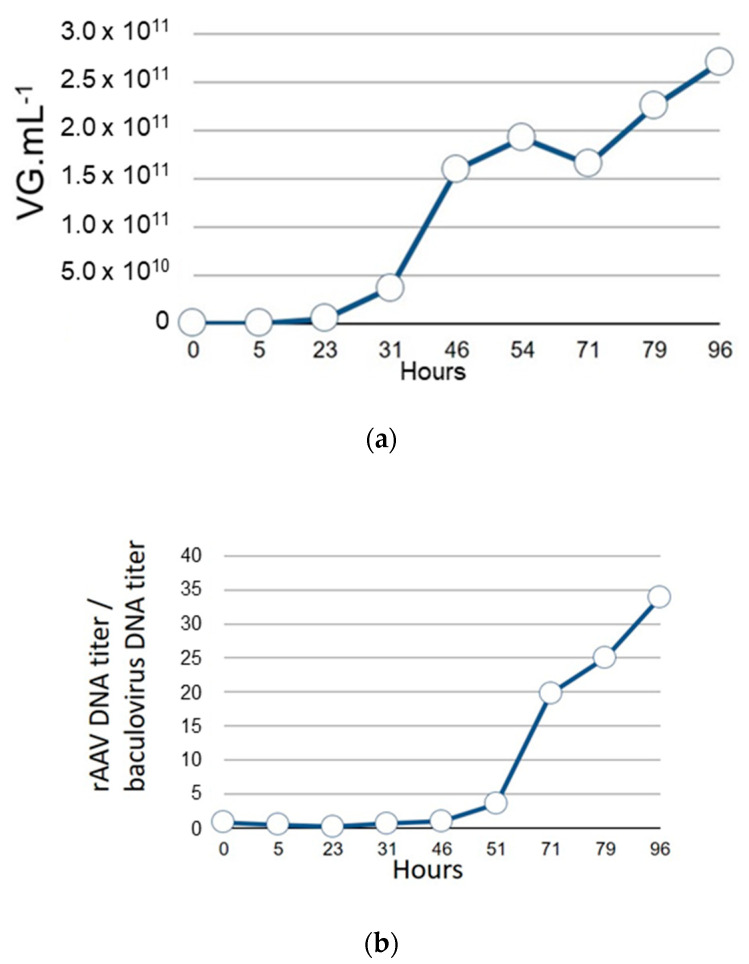
(**a**) Time course of replication of baculovirus DNA during rAAV8-γSGC production in the dual baculovirus system with one baculovirus-encoding recombinant rAAV8-γSGC transgene inserted at the Tn7 site and the second baculovirus encoding cap8 gene at the *egt* locus and rep2 gene inserted at the Tn7 site. Total MOI was 0.1 (*n* = 2). Follow-up was performed for 96 h. Baculovirus DNA copy number, based on DNA Polymerase qPCR amplicon measurement, is expressed in number of copies per mL of cell culture. (**b**) Ratio of rAAV8-γSGC gene copy number to baculovirus DNA copy number, measured by qPCR of the baculovirus DNA polymerase gene amplicon, during rAAV8-γSGC production.

Prior to the construction of the single baculovirus system for rAAV production, we evaluated, in the dual baculovirus system [20], the kinetics of rAAV genome replication compared to the replication of the baculovirus genome. Indeed, we had previously observed that the wt-AAV2 genome inserted in the baculovirus as “rAAV transgene” underwent excision from the baculovirus genome due to the early Rep78 protein expression originating from the p5 promoter [18]. In the dual baculovirus expression system [20], the Rep protein expression is driven from the very-late baculovirus *polyhedrin* gene promoter and should be expressed at the very-late stage of infection. Meanwhile, the replication of the baculovirus DNA along with the synthesis of the budded virus (BV) particles should happen earlier. To verify this, we used the dual baculovirus production system to monitor the amplification kinetics of both baculovirus and rAAV genomes. During production of rAAV using the dual baculovirus system with infection performed with an MOI of 0.1 (0.05 for each baculovirus), amplification of the baculovirus genome was initiated between 5 and 23 h pi with an increase in the level of baculovirus DNA detected 23 h pi with 4 × 10^10^ vg mL^−1^ of bulk product (Figure 2a). Between 23 h and 46 h pi a sharp increase in replication level of baculovirus genome was observed with 1.5 × 10^11^ vg mL^−1^ 46 h pi. Following this time point, up to the end of the production 96 h pi, the level of replicated baculoviral DNA plateaued at 2.5 × 10^11^ vg mL^−1^. By comparison, the rAAV genome replication started to be detected between 46 h and 51 h pi with three times more rAAV genomes detected compared to the baculovirus genome level (Figure 2b). At 71 h pi, 20 times more copies of rAAV genome were detected compared to baculovirus genome copies. rAAV genome copy numbers increased steadily, reaching 34 times more genome copies compared to baculovirus genome copies at the end of the kinetics measurements. This decoupling of baculovirus genome amplification compared to rAAV genome replication would mean that both the rAAV genome and polyhedrin promoter-driven Rep expression cassette can be present in the same recombinant baculovirus.

### 3.3. Generation of the Monobac and Production of rAAV Vectors

**Figure 3:**
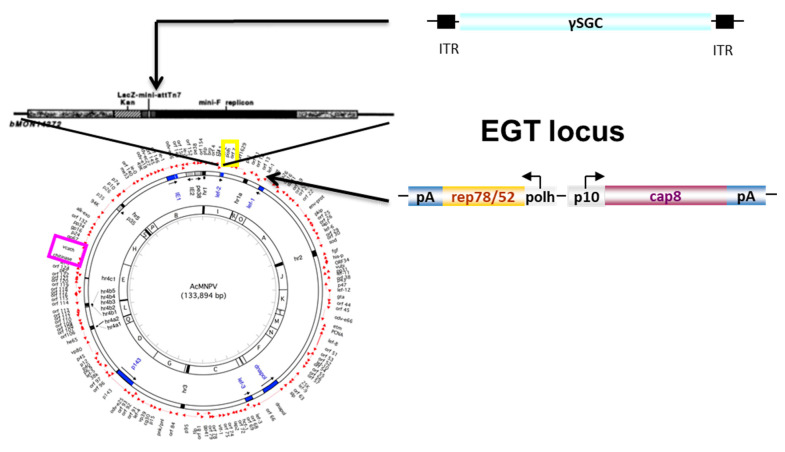
Schematic representation of the Monobac system. Homologous recombination is performed at the *egt* locus using plasmid pKD46 encoding the red recombination genes [28] in DH10 bac cells. The *cat* gene used to select positive clones can then be removed using a Cre-encoding plasmid (this study) leading to a Cre-insensitive lox72 DNA scare [24]. With this method, additional genes can be inserted or removed from the AcMNPV bacmid genome, like the deletion of AcMNPV *cathepsin-chitinase* genes [35]. pMON7124 [26] is then used to promote recombination at the Tn7 site of the bacmid, using a donor plasmid encoding the rAAV transgene and flanked by the Tn7L and R sequences.

Based on the bacmid technology [26], we inserted the rep2-cap8 expression cassette [20] into the *ecdysteroid glucosyl transferase* gene (*egt*) of the *Autograph californica* multi nuclear polyhedrosis virus (AcMNPV) (Figure 3). The baculovirus *egt* gene plays a role in preventing molting and pupation of infected caterpillar larvae [33]. The *egt* gene is also involved in virus spreading by promoting larvae to move to higher positions on plants through phototaxy [34]. This gene is dispensable in cell culture and is not involved in budded virus (BV) genesis [27]. The rAAV expression cassette encoding γSGC gene was then transferred into the Tn7 site of the modified bacmid genome [26].

**Figure 4:**
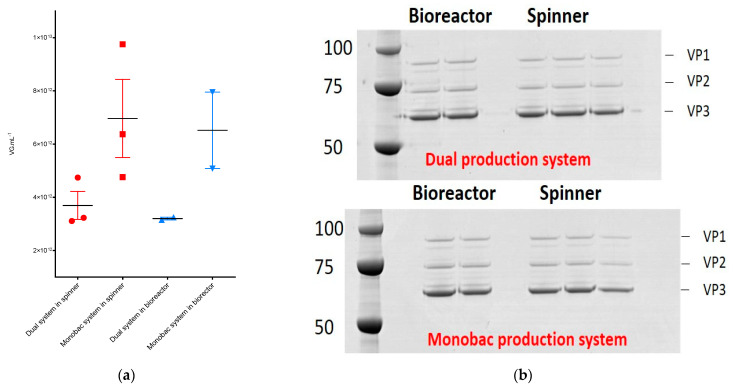
Comparison of rAAV8-γSGC productivity between the dual baculovirus and the Monobac systems in spinner and bioreactor cultures. (**a**) Purified rAAV8-γSGC produced in Spinner (in red) (*n* = 3) and bioreactor vessels (in blue) (*n* = 2); comparison between the dual baculovirus (●, ▲) and Monobac (■, ▼) systems. rAAV8-γSGC titers are expressed in vg mL^−1^. Each data label represents an individual production run. Horizontal black lines represent the average titers obtained. Error bars represent standard deviation. (**b**) Protein profiles of each rAAV8-γSGC run after vector purification are visible on Western blots using B1 progen antibody for the detection of AAV VP proteins. 5 × 10^9^ vg of purified vector have been deposed per lane. The SDS-PAGE gel (top) represents rAAV8-γSGC produced using the dual baculovirus production system. The SDS-PAGE gel (bottom) represents rAAV8-γSGC produced using the Monobac production system.

We performed production and purification of rAAV8-γSGC at spinner scale in parallel using the dual and the single baculovirus system. The average yield of rAAV8 vectors produced with the dual baculovirus system resulted in an average of 3.7 × 10^12^ vg mL^−1^ (+/-SD (standard deviation) 9.1 × 10^11^ vg mL^−1^), while the Monobac produced an average rAAV8-γSGC titer of 7.0 × 10^12^ vg mL^−1^ (+/-SD 2.5 × 10^12^ vg mL^−1^) (Figure 4a). Protein profiles of purified rAAV displayed similar protein bands and intensities when performed at constant vg levels (Figure 4b). Productions performed in 2 L bioreactors showed a similar difference in purified rAAV yields with an average of 3.2 × 10^12^ vg mL^−1^ (+/-SD 7.1 × 10^10^ vg mL^−1^) with the dual baculovirus system versus 6.5 × 10^12^ vg mL^−1^ (+/-SD 2.0 × 10^12^ vg mL^−1^) for the Monobac system. Thus, the Monobac system resulted in an overall increase by a factor of two in productivity of rAAV8-γSGC, while conserving similar VP protein profiles compared to the dual baculovirus system as observed by SDS-PAGE followed by Coomassie staining and the Western blot of capsid proteins (Figure 4b).

**Figure 5:**
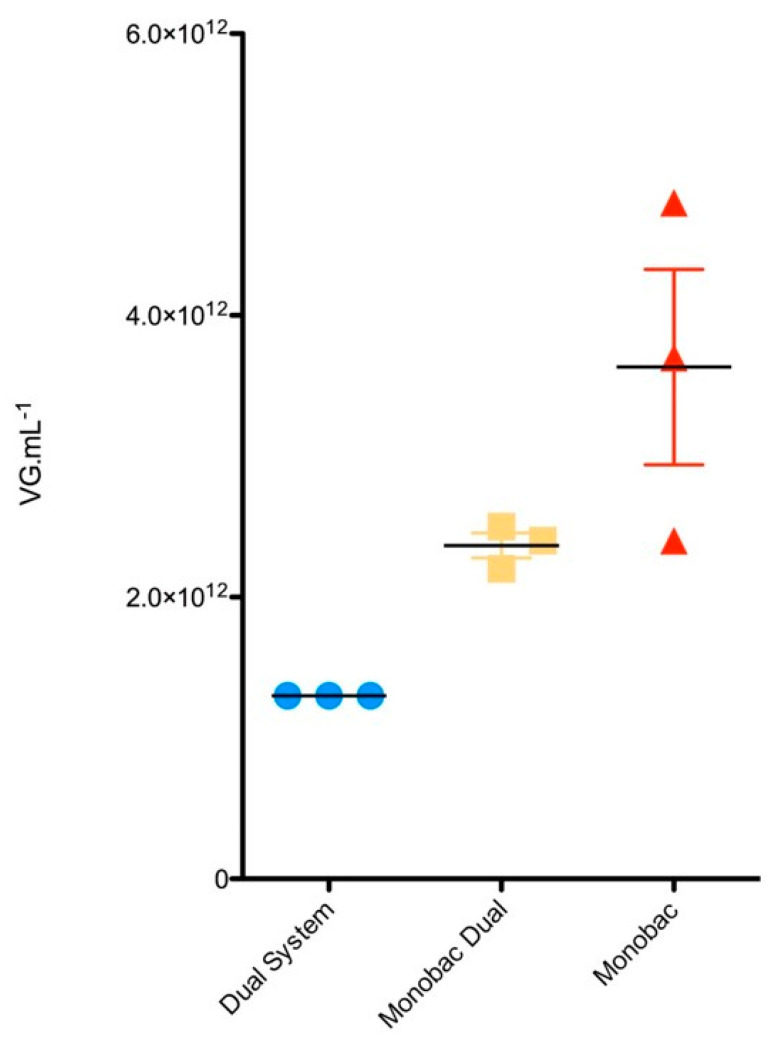
Impact of rep2-cap8 cloning position in the baculovirus genome on rAAV production. rAAV8-γSGC titers in bulk are expressed in vg mL^−1^ of cell culture and represent the mean of 3 production runs. Error bars represent standard deviation. “Dual system” represents production performed using the dual-baculovirus rAAV production system [20]. “Monobac dual” represents productions performed with one baculovirus encoding rAAV8-γSGC transgene at Tn7 site while the second baculovirus encodes rep2-cap8 expression cassette from the *egt* locus. “Monobac” represents productions performed using the single baculovirus with rAAV8-γSGC genome inserted at the Tn7 site and rep2-cap8 expression cassette cloned at the *egt* locus.

We then wanted to determine whether the productivity increase measured for the Monobac over the dual baculovirus production system was due to the cloning of the rep2-cap8 expression cassette at the *egt* site in comparison to the Tn7 and/or from the higher proportion of cells infected by a single baculovirus over a co-infection. rAAV8-γSGC produced with dual baculovirus infection, with an rep2-cap8 expression cassette and rAAV both inserted at the Tn7 site of the baculoviruses, led to an rAAV titer in the bulk of 1.3 × 10^12^ vg mL^−1^ (Figure 5, “Dual System”). Production using two baculoviruses with a rep2-cap8 expression cassette inserted at the *egt* locus of the first baculovirus and rAAV-γSGC transgene inserted at the Tn7 site of the second baculovirus led to an average bulk titer of 2.4 × 10^12^ vg mL^−1^ (Figure 5, “Monobac Dual”). Productions using one baculovirus with a rep2-cap8 expression cassette inserted at the *egt* locus and a rAAV γSGC transgene inserted at the Tn7 site led to an average titer of 3.6 × 10^12^ vg mL^−1^.

**Figure 6:**
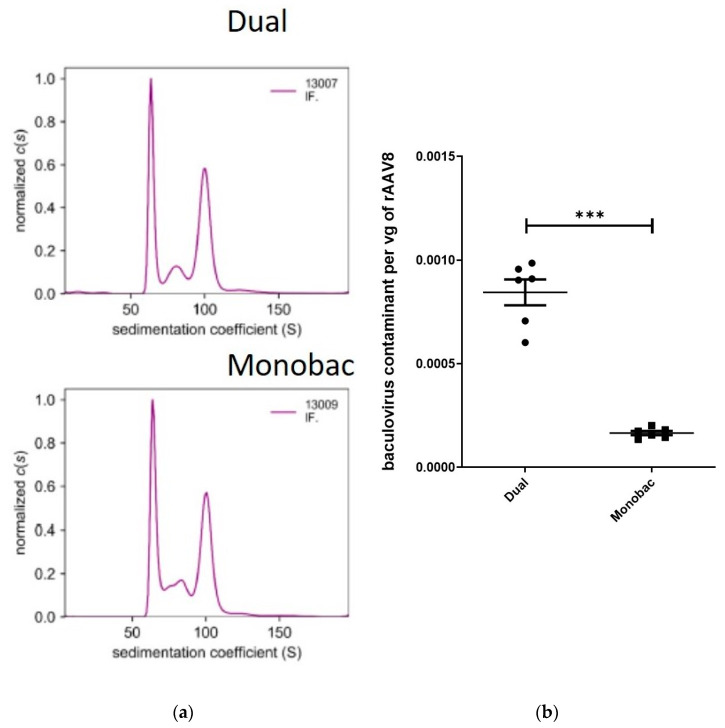
Comparison of rAAV8-γSGC production performed using the dual baculovirus and Monobac systems. Comparison of rAAV8-γSGC production performed with dual baculovirus (Dual WT) and Monobac systems (MB WT) analyzed by (**a**) analytical ultracentrifugation sedimentation coefficient results for purified rAAV8-γSGC produced with dual baculovirus and Monobac production systems (shown are the data obtained by interference monitoring). Values have been normalized to c(s). Empty rAAV particles sediment between 64 and 66 S, full rAAV8 particles have sedimentation values comprised between 99 and 103 S. Values between 76 and 90 S correspond to capsids partially filled with a genome size between 1 and 3 kb. The values < 60 S correspond to impurities smaller than rAAV particles that could be free proteins or DNA. (**b**) qPCR ratio between baculovirus DNA polymerase amplicon per purified rAAV8-γSGC vg titers (*t*-test, *p* = 0.002, ***).

Vectors produced either with the Monobac or dual baculovirus production systems displayed equivalent levels of VPs proteins when observed by SDS-PAGE and Western blot. To further characterize rAAV8-γSGC production performed using either the dual or Monobac systems, full and empty capsid ratios were analyzed using analytical ultra centrifugation (AUC): we observed 42% of full particles using the dual baculovirus system, while we observed 36% of full particles for the Monobac system. Empty capsid levels were 43% and 44%, respectively, while partially filled capsids represented 14% and 20%, respectively (Figure 6a).

Interestingly, an evaluation of contaminating baculovirus DNA using qPCR on the baculovirus DNA polymerase gene produced a five-fold reduction (*t*-test, *p* = 0.002) of contaminating baculovirus DNA in favor of the Monobac system (Figure 6b). This important difference in contaminating DNA levels may reflect the effect of Sf9 cells infected only with the baculovirus rep2-cap8, which are unable to fill the rAAV capsids with the rAAV transgene in the absence of co-infection with the baculovirus encoding the rAAV transgene.

**Figure 7:**
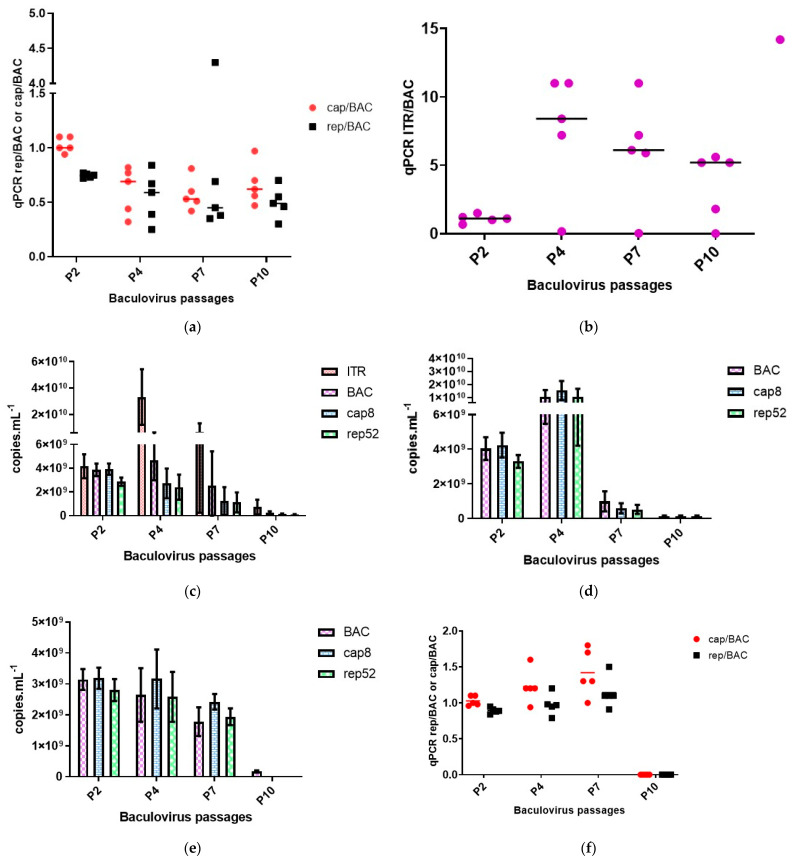
Genetic stability of Monobac- γSGC over 10 passages in culture flasks. We studied the genetic stability of the Monobac with and without the rAAV- γSGC transgene and the rep2-cap8 baculovirus from the dual rAAV production system. The *x*-axis represents the number of passages. Results were obtained by qPCR. The ITR sequence allows the follow-up of the rAAV-γSGC transgene cloned in the Monobac genome and, at the same time, the rAAV-γSGC produced during the infection. The BAC sequence quantifies the baculovirus DNA polymerase gene. The Rep52 and cap8 sequences are used to quantify the rep2-cap8 expression cassette inserted in the *egt* gene in the Monobac and in the Tn7 site of the baculovirus in the dual-rAAV production system. Results are expressed either as copies per mL or as ratio of either ITR, cap8, and rep52 sequences in comparison to the BAC sequence. Individual clones are represented with the horizontal line indicating the mean or data are shown as histogram bars with the SD. The *x*-axis indicates the number of passages. P2 represents the baculovirus seed stocks used in this experiment, while P4, P7, and P10 represent the baculovirus stocks at passages 4, 7, and 10. (**a**) Monobac-γSGC, stability of the rep2-cap8 expression cassette. We followed the ratio between the *rep2* gene and the *cap8* gene over the baculovirus *DNA polymerase* gene. (**b**) Monobac-γSGC, rAAV versus baculovirus productivity. We followed the number of rAAV particles produced per baculovirus through the ratio between ITR-based qPCR and baculovirus DNApol qPCR. (**c**) Monobac-γSGC, copies per mL of ITR BAC, rep and cap sequences. Copies per mL of the ITR, cap8, rep52 and BAC sequences. (**d**) Monobac without any rAAV transgene. Copies per mL of cap8, rep52, and BAC sequences. (**e**) The rep2-cap8 containing baculovirus of the dual rAAV production system. Copies per mL of cap8, rep52, and BAC sequences. (**f**) The rep2-cap8-containing baculovirus of the dual rAAV production system; stability of the rep2-cap8 expression cassette. We followed the ratio between the rep2 gene and the cap8 gene over the baculovirus DNA polymerase gene.

A critical point is the genetic stability of the baculoviruses during the amplification of virus stocks and their ability to conserve the transgenes of interest. To investigate the genetic stability of the baculovirus genomes, we performed viral passages starting with viral stocks amplified for two passages following plaque purification of the baculovirus clones. The baculoviruses were passaged on Sf9 cells cultured in flasks to reach ten serial passages. At passages 4, 7, and 10, qPCR analysis were performed on either baculovirus DNA polymerase gene, AAV *rep* and *cap* genes and on the ITR region of the rAAV transgene to measure the baculovirus and rAAV titers when the Monobac was used. Two data sets are presented for each baculovirus constructs. Either the qPCR titers of each amplicon, reflecting the passage effect of each baculovirus stock and thus the global loss of functional baculovirus particles. The second set of figures presents the ratio between the recombinant transgenes, either rep, cap, or ITR rAAV transgene, in relation to the baculovirus DNA polymerase essential gene. These ratios represent the genetic stability of the heterologous genes inserted in the baculovirus genome and at which speed the baculovirus eliminates them from its genome. Starting the study with the Monobac encoding the rAAV-γSGC transgene, we characterized five clones. The baculovirus stock used for this experiment displayed an average ratio of 1.08 for the cap/BAC, 0.75 for the rep/BAC ratio (Figure 7a, column P2), and 1.09 for the ITR/BAC ratio (Figure 7b, column P2) with all individual clones displaying similar ratios. This indicates a homogenous stock of each baculovirus clones, with all baculovirus containing the *rep* and *cap* genes at the *egt* locus and the rAAV γSGC transgene at the Tn7 site. At the fourth passage of the Monobac encoding the rAAV transgene, the harvest was performed four days post-infection. At this time, baculoviruses were amplified in the Sf9 cell culture and produced rAAV γSGC. It was visible when observing the ITR/BAC ratio, with an average ratio of 7.6 (Figure 7b, column P4). At this passage, one clone lost almost completely the rAAV γSGC cassette from its genome with a ratio ITR/BAC of 0.025. The average rAAV γSGC titer obtained was 3.32 × 10^10^ vg mL^−1^ in comparison to a baculovirus titer (evaluated using the BAC amplicon qPCR) of 4.68 × 10^9^ vg mL^−1^ (Figure 7c, column P4). At passage 7, the average rAAV γSGC titer obtained was 1.84 × 10^10^ vg mL^−1^ in comparison to a baculovirus titer (evaluated using the bac amplicon qPCR) of 2.52 × 10^9^ vg mL^−1^ (Figure 7c, column P7). The clone already observed at passage 4 displaying lower rAAV productivity with an ITR titer of 7.8 × 10^8^ vg mL^−1^ and a BAC titer of 4.8 × 10^9^ vg mL^−1^ continued to drop with an ITR titer of 1.9 × 10^8^ vg mL^−1^ while the baculovirus titer measured using the BAC amplicon was 7.9 × 10^9^ vg mL^−1^. Overall, the ratio between ITR/BAC amplicon was more dispersed between the individual clones at passage 7 (Figure 7b, column P7). At passage 10, the rAAV productivity dropped compared to the previous passages reaching an average of 7.26 × 10^8^ vg mL^−1^ for the ITR amplicon (Figure 7c, column P10), while the average baculovirus titer was of 2.44 × 10^8^ vg mL^−1^. The average ITR/BAC amplicon titer was of 3.56 (Figure 7b, column P10). Through this baculovirus genetic stability assay over passages, we can observe two phenomena. The passage effect resulted in the loss of functional baculoviruses reflected with the reduction in the bac qPCR titer over the passages. The ITR/BAC ratio reduction over the passages indicates that the ITR cassette clones at the Tn7 site of the baculovirus was more prone to excision than the rep-cap cassette, from the baculovirus genome. Indeed, by comparison, the rep-cap expression cassette ratio compared to the BAC amplicon stayed constant overall over the baculovirus passages (Figure 7a), indicating a stability of the rep-cap expression cassette when inserted in the *egt* locus of the baculovirus genome. The Monobac baculovirus without any rAAV cassette present at the Tn7 site also displayed the passage effect with a drop of the BAC titer visible from passages 4 to 10 (Figure 7d). The stability of the rep-cap expression cassette was equally conserved with an overall stable ratio between the rep/BAC and cap/BAC qPCRs over the passages. The baculovirus rep-cap, with the expression cassette inserted in the Tn7 site of the baculovirus, is also subject to the passage effect with a decrease in baculovirus qPCR titers over passages (Figure 7e, column P10). However, in the Monobac system, at the same passage, the rep-cap expression is still detectable and present in the genome of the functional baculovirus (Figure 7a, column P10 versus Figure 7f, column P10). These results highlight the superior genetic stability of an expression cassette (e.g., Rep-cap expression cassette) when inserted in the *egt* locus over the Tn7 site of the bacmid as previously described in another baculovirus-based expression system [31].

### 3.4. In Vivo Performance of rAAV8 Vectors Produced Using the Monobac

**Figure 8:**
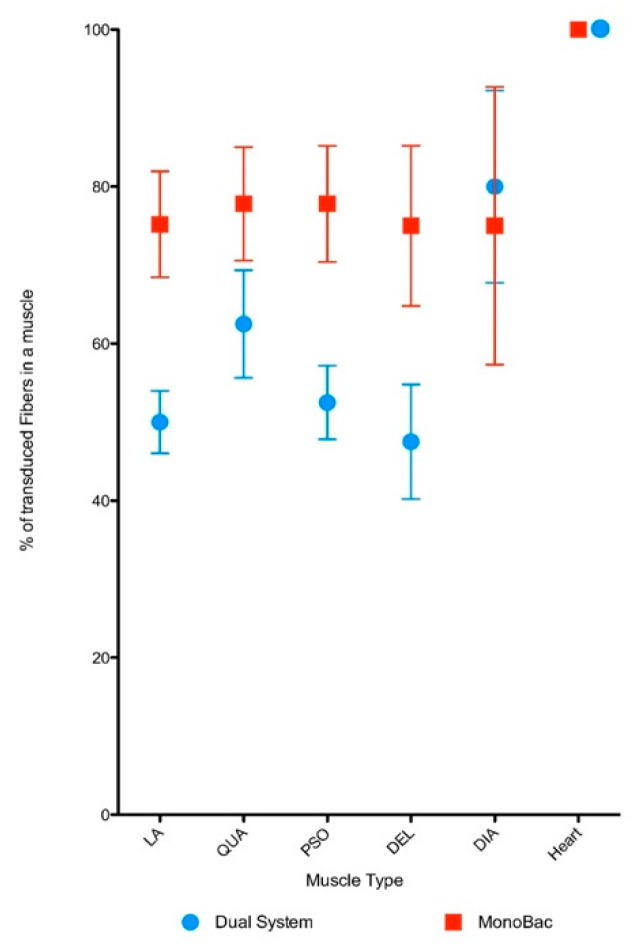
In vivo evaluation of rAAV8-γSGC vector potency. Quantification of muscle fibers positive for γSGC protein expression presented as percentage of muscle fibers (+/-SD) positive for γSGC protein expression 28 days after transduction with rAAV8-γSGC produced using the Monobac (red dots) or the dual baculovirus production (blue dots) system at a dose 5 × 10^12^ vg kg^−1^ of vector were injected in the tail vein of the γSGC mouse model following delivery of AAV produced with dual baculovirus and Monobac systems. The muscles studied were deltoid, diaphragm, anterior loge, psoas, quadriceps, and heart.

To evaluate the potency of the rAAV8-γSGC produced using the Monobac, 5 × 10^12^ vg kg^−1^ of vector were injected in the tail vein of the γSGC mouse model. Muscle sections were studied to detect expression of the γSGC protein. Loge Anterior muscle fibers were transduced with an average of 50% with rAAV8 vectors produced in the dual baculovirus system while 75% of the fibers were transduced with the rAAV8-γSGC produced using the Monobac (Figure 8). This increase in the level of transduction was observed in comparable ranges for quadriceps (62.5% for the dual baculovirus versus 77.5% for the Monobac product), psoas (50% versus 77.5%), and deltoid muscles (47.5% versus 75%). Transduction level of diaphragm muscles was in the same range with 80% for rAAV8-γSGC produced with the dual baculovirus system compared to 75% with the Monobac system. Cardiac muscle cells were fully transduced with rAAV8-γSGC produced in both systems. The slight increase in transduction level observed for striated muscles of mice injected with rAAV8-γSGC produced with the Monobac was observed for all striated muscles studied except for the diaphragm muscle. The high level of transduction of cardiac muscle cells has been described for AAV8 vectors in mice at higher doses [32] previously, although expression cassette and quantification methods were different. Overall, we can conclude that rAAV8-γSGC vectors produced with the Monobac are at least as efficient as the one produced with the dual baculovirus system.

## 4. Discussion

We successfully developed a single baculovirus for the production of rAAV, which fulfils the needs of industrial producers to have simplified large-scale and cost-effective production processes. Starting from the material developed by Smith et al. [20], we combined the rep2-cap8 expression cassette and the recombinant rAAV cassette into a single baculovirus. We observed a two-fold increase in rAAV titers when using the Monobac in comparison to the dual baculovirus production system. This increase in titer level reflects what is observed when rAAV is produced with a dual system encoding fluorescent protein genes where only 40% of the cells were co-infected by both types of baculoviruses.

This gain in productivity could also be associated with the cloning position of the rep-cap expression cassette, with the *egt* gene being a more favorable context over the Tn7 site (Figure 5, Dual System versus Monobac Dual) for the *rep* and *cap* gene expression. This locus was also favored for the expression of heterologous proteins by Noad et al. [27]. The rAAV produced with either the Monobac or the dual baculovirus system displayed similar profiles of full and empty particles when analyzed by AUC. However, rAAV produced with the Monobac system displayed a five-fold reduction in the level of baculovirus contamination levels compared to the dual baculovirus production system. This higher level of contaminating DNA would potentially arise from the Sf9 cells infected only with the baculovirus encoding the rep-cap genes. When measured, 23% of Sf9 cells were only infected with the baculovirus encoding the rep-cap genes. Proportionally, the dual baculovirus system increased the level of contaminating DNA by five-fold. This would suggest that, in the absence of the baculovirus encoding the rAAV transgene, the AAV replicases are more prone to package the contaminating baculovirus DNA.

This lower level of contaminating baculovirus DNA found in the rAAV could not be observed by AUC. This probably indicates relatively short baculovirus-contaminating DNA sequences packaged or associated to rAAV capsids.

When developing the Monobac system, we were concerned that the expression of Rep78 would lead to rAAV genome excision from the baculovirus genome during amplification of baculovirus stocks. Indeed, excision of rAAV vector transgene from producer plasmid has been characterized in mammalian cells when large Rep proteins are expressed [36]. In the Monobac system, we showed the delayed *rep78* gene expression performed under very-late baculovirus *polyhedrin* promoter did not lead to the loss of the recombinant rAAV transgene in the recombinant baculovirus. Thus, single baculovirus production system using this strategy may restrict the choice of promoter that could be used to drive the expression of the Rep proteins. An immediate early baculoviral promoter such as the short version of OpIE1 (Immediate early 1 promoter from *Orgyia Pseudotsugata* MNPV) as used in the original work of Urabe et al. [17] would probably result in excision of the recombinant AAV genome as occurs for plasmid rescue in mammalian cells [36]. As a consequence, the choice of a promoter compatible with the Monobac strategy may have to be reduced to late and/or very-late expression patterns [37]. In another work, we have shown the excision of a wild type AAV2 cassette cloned in the baculovirus genome [21] resulting from the early expression of the Rep78 protein from the p5 promoter. In this case the excision from the baculovirus genome was happening following transfection of the bacmid DNA containing the wt-AAV2 cassette. By comparison, the loss of the rAAV-γSGC transgene from the baculovirus genome happened at a much slower rate, reflecting the well-known “passage effect” of the baculoviruses [38,39,40].

The Monobac system becomes particularly enticing in GMP conditions where only one stock of baculovirus needs to be amplified and characterized for the Master Virus Bank. Mixing two unevenly titered stocks during infection is inherently stochastic; thus, single baculovirus infection adds robustness to the production process.

From a functionality point of view, both the Monobac system and the OneBac system [41] are equivalent and are able to produce comparable vector levels. However, the main difference relates to the generation of a new baculovirus when the rep or cap sequences have to be changed or when another rAAV vector has to be produced. In the case of the OneBac system, the desire to produce another AAV serotype (either with another wild type or chimeric capsid) or an rAAV vector with a modified Rep protein requires the development of new producer cell line whose development and characterization needs more investment and efforts compared to the development of a new baculovirus.

Wu et al. [42] presented a system closely related to the Monobac in which the expression cassette containing the rep and cap genes and the rAAV genome is inserted at the Tn7 of the bacmid system. The authors have also been able to efficiently produce rAAV vectors. Although we did not compare both systems side-by-side, we suspect that the use of such a large cassette at the Tn7 site may promote genetic instability, making the Monobac system more likely to be stable following amplification passages. Another difference in the Wu et al. system is that the rAAV transgene cassette is flanked by the *rep* and *cap* genes. We have shown in this study that the integrity of the ITR has a significant impact on the production of rAAV vectors. Specifically, the DNA flanking the ITRs can represent up to 1% of the contaminating DNA in comparison to the rAAV vg titer, depending on the ITRs used [43]. The higher level of DNA packaging in the rAAV capsid comes from sequences flanking the ITRs, which has also been shown by Penaud-Budloo et al. [44]. In the Wu et al. design, this may potentially lead to higher levels of packaging of *rep* and *cap* contaminating sequences. The *rep* and *cap* genes are driven by *polyhedrin* and *p10* baculovirus promoter, which are dependent on the baculovirus RNA polymerase for their transcription [45] and thus should not be active in the mammalian cells following rAAV delivery. Still, one cannot preclude the possibility of recombination leading to functional rep or cap sequences. It is similarly possible that promoter activity arising from the ITRs [44] could lead to basal expression of *rep* or *cap* following rAAV delivery in vivo. For these reasons, we consider the Monobac as a safer approach to produce rAAV vectors in insect cells.

In conclusion, we presented here a novel single baculovirus system for the production of high titer functional rAAV vectors.

## 5. Patents

This work has led to the patent WO/2013/014400.

## Figures and Tables

**Table 1:** Primer used in this study.

Primer	Sequence 5′ to 3′	Purpose
EGT-lox-F	TTACGGTCGTCAAGCCCAAACTGTTTGCGTATTCAACTAAAACTTATTGCGGTAATATCACTACCGTTCGTATAGCATACATTATACGAAGTTATAATAGGAACTTCATTTAAATGGCGC	PCR product for rep2-cap8 and cap8 cloning at egt
EGT-SV40-R	TCCCGGCTTCCAAGGCCTCGTCGCTCGATTGTAGTCCGCCTTGCGTAATAAACGCCGCCATTTTTTTATGACGCAGCACGG CAGACATGATAAGATACATTGATGAGTTTG	PCR product for rep2-cap8 cloning at egt
EGT-p10-R	TTACGGTCGTCAAGCCCAAACTGTTTGCGTATTCAACTAAAACTTATTGCGGTAATATCACTACCGTTCGATATAGCATACATTATACGAAGTTATAATAGGAACTTCATTTAAATGGCGC	PCR product for cap8 cloning at egt
EGT-F	ATGACTATTCTCTGCTGGC	KI verification
EGT-R	ATTGGCCGTGTTTCCTAC	KI verification
M13 PUC F	CCAGTCACGACGTTGTAAAACG	Verification of transposed bacmids
M13 PUC R	AGCGGATAACAATTTCACACAGG	Verification of transposed bacmids
Genta	AGCCACCTACTCCCAACATC	Verification of transposed bacmids
ITR-F	CTCCACTAGGGGTTCCTTG	QPCR rAAV ITR
ITR-R	GTAGATAAGTAGCATGGC	QPCR rAAV ITR
ITR-P	[FAM] TAGTTAATGATTAACCC [MGB-NFQ]	QPCR rAAV ITR
BAC-F	ATTAGCGTGGCGTGCTTTTAC	QPCR DNApol AcMNPV
BAC-R	GGGTCAGGCTCCTCTTTGC	QPCR DNApol AcMNPV
BAC-P	[VIC] CAAACACGCGCATTAACGAGAGCACC [TAMRA]	QPCR DNApol AcMNPV
Rep52-F	GCCGAGGACTTGCATTTCTG	QPCR rep2
Rep52-R	TCGGCCAAAGCCATTCTC	QPCR rep2
Rep52-P	[VIC] TCCACGCGCACCTTGCTTCCTC [TAMRA]	QPCR rep2
Cap8-F	TTCTGCAGCTCCCATTCAATT	QPCR cap8
Cap8-R	TCAACCAGTCAAAGCTGAACTCTT	QPCR cap8
Cap8-P	[VIC] CCACGCTGACCTGTCCGGTGC [TAMRA]	QPCR cap8
